# Dietary replacement of soybean meal with black soldier fly larvae meal in juvenile *Labeo rohita* and *Catla catla*: Effects on growth, nutritional quality, oxidative stress biomarkers and disease resistance

**DOI:** 10.1371/journal.pone.0294452

**Published:** 2023-11-20

**Authors:** Shafaq Fatima, Ayesha Afzal, Hamna Rashid, Saba Iqbal, Rosheen Zafar, Komal Khalid, Ayman Rauf, Maryam Majeed, Aqsa Malik, Chris G. Carter

**Affiliations:** 1 Department of Biological Sciences, Purdue University Fort Wayne, IN, United States of America; 2 Department of Zoology, Lahore College for Women University, Jail Road, Lahore, Punjab, Pakistan; 3 Institute for Marine and Antarctic Studies (IMAS), University of Tasmania, Hobart, TAS, Australia; Sher-e-Kashmir University of Agricultural Sciences and Technology of Kashmir, INDIA

## Abstract

This experiment aimed to investigate the effects of partial substitution of crude protein from soybean meal (SBM) with black soldier fly (*Hermetia illucens*) larvae meal (BSFLM) in juvenile rohu (*Labeo rohita*) and catla (*Catla catla*). Four isonitrogenous diets (23% crude protein) were formulated to replace 0% (T0), 40% (T40), 80% (T80) and 100% (T100) crude protein from SBM with BSFLM. Triplicate groups of each species (10 fish per replicate) were fed in an eight week growth experiment. After final sampling (n = 20 fish per dietary group), the remaining fish were exposed to bacterial (*Staphylococcus aureus*) challenge (0.80 CFU/ml) for 15 days. Rohu fed with BSFLM substituted diets showed significantly higher growth and feed conversion ratio as compared to those in T0. Catla fed with BSFLM substituted diets showed slightly higher growth indices. The growth response of rohu to BSFLM substitution was better than that noted in catla in all groups. The chemical composition, amino acids and fatty acids profile, haematological and biochemical parameters, levels of liver function enzymes measured in T0, T40, T80 and T100 were similar between four dietary groups in both species. However, the maximum value of cholesterol and triglycerides were noted in T100 both in catla and rohu. The values of lauric acid, α-linolenic acid, decosahexanoic acid, n3:n6 fatty acids ratio progressively increased with dietary increase of BSFLM in both species. At end of the growth experiment, the levels of catalase, superoxide dismutase and lysozyme increased linearly with the inclusion of BSFLM in both species while malondialdehyde showed similar values between different groups. However, catalase, and superoxide dismutase increased (T0<T40<T80<T100) in both rohu and catla after exposure to bacterial challenge while malondialdehyde remained almost the same. These biomarkers indicate that substitution of SBM with BSFLM up to 100% improved disease resistance in both species against gram-positive bacteria.

## 1. Introduction

In 2020, a historic global aquaculture production of 122.6 million tons, valued at USD 281.5 billion, was documented. Asia continued to be the dominant player in world aquaculture, contributing 91.6% of the total production, while 93.5% of fish farmers worldwide were concentrated in Asia. The primary category of species produced in 2020, comprising carps, barbels, and other cyprinids (1.9 million tons), accounted for 18% of the global production. Rohu (*Labeo rohita*) and catla (*Catla catla*) ranked as the 5^th^ and 9^th^ most produced species, respectively, in global cyprinid inland aquaculture by production [1]. Besides increasing production, prioritizing the improvement of feed for these species, which constitute the largest share of aquafeed, is a key focus for the aquaculture industry.

Globally, soybean stands out as the primary source of plant protein for both livestock and fish feed. In the period of 2021/2022, numerous countries collectively imported approximately 162.76 million metric tons (MMT) of soybean. China led the pack as the top importer, receiving around 97 MMT that year [2], both on a global scale and within the region. Other Asian countries collectively imported an estimated 43 MMT of soybean [2, 3]. This heavy reliance on soybean imports in the region raises concerns about the potential vulnerability of its sustainable supply in the future. Additionally, there are ongoing worries about the environmental repercussions associated with its production. Furthermore, soybean is deficient in certain crucial amino acids, contains anti-nutritional factors [4], and faces resistance to its use, particularly in countries averse to genetically modified varieties. Hence, a critical area of focus for reshaping Asian aquaculture involves exploring novel protein sources that can be cultivated locally, thereby securing a sustainable protein supply chain and mitigating the expenses and uncertainties tied to imports [5]. The larvae of the black soldier fly (*Hermetia illucens*) (BSFL) have garnered attention for their circular economy attributes and are swiftly emerging as an alternative. They present a more environmentally friendly, cost-effective, and sustainable option for fish feed [6]. Moreover, BSFL cultivation does not demand an extensive utilization of land and water resources, distinguishing it from plant-based alternatives. Considering the challenges posed by climate change and the imperative of environmental sustainability, BSFL is increasingly being considered as a promising candidate for aquafeed protein.

Black soldier fly (*Hermetia illucens*) larvae (BSFL) meal is rapidly emerging alternative protein source which is economical, have lesser environmental impact and a sustainable option for aquafeed sector [6]. Production of BSFLM does not require large land and water resources unlike plants therefore, it can be a suitable candidate for aquafeed protein among the challenges of climate change and sustainability of environmental resources. The chemical composition, amino acid, fatty acid, and minerals contents of BSFL is controlled by the type of consumed food material (mixed food, fruit, fish, seafood, bread and vegetable waste, livestock manure) [7, 8]. The chemical composition of BSFL comprises of 50%-60% of crude protein, 30–35% of lipids, and amino acids [9, 10], 58%-72% saturated fatty acids and 19%-40% mono and polyunsaturated fatty acids [7, 11] depending upon the type of ingested food. Moreover, BSFL has its immunomodulatory importance due to presence of antimicrobial peptides, lauric acid, and chitin [12–14]. Lauric acid has particularly showed potent antibacterial and antiviral properties with the highest efficacy against gram-positive bacteria [13]. This anti-microbial and anti-viral role of BSFLM needs to be investigated further in different fish species against local pathogens which cause massive economic loss. An excellent nutrient profile, short breeding time period (hatching: 4 days, larvae stage: 18 days), easy handling [6], cheap raw feeding material, selection of feeding material as per feed requirement, makes BSFL a suitable alternative protein source for aquafeed. BSFL is already being cultured at a large scale in different countries to fulfill the protein demand of poultry and pet feed [15]. In Europe, EU directive (EU Regulation No.: 2017/893) [16] in effect from 2017, has authorized the use of BSFL in fish feed.

Several studies have been reported on the replacement of fishmeal and fish oil by BSFL meal (BSFLM) in carnivorous fish species and shrimp, including rainbow trout (*Oncorhynchus mykiss*) [17, 18], meagre (*Argyrosomus regius*) [19], turbot (*Psetta maxima*) [11], Atlantic salmon (*Salmo salar*) [20–22], Japanese seabass (*Lateolabrax japonicus*) [23], Siberian sturgeon (*Acipenser baerii*) [24], African catfish (*Clarius gariePinus*) [25], largemouth bass (*Micropterus salmoides*) [26], eel (*Monopterus albus*) [14], European sea bass (*Dicentrarchus labrax*) [27, 28], and pacific white shrimp (*Litopenaeus vannamei*) [29]. Most of these studies reported that different levels of substitution of fishmeal (100% - 75%) by BSFLM did not impair growth, chemical composition, nutritional quality, immune response, and digestive performance. The limited studies on herbivore and omnivore fish species include yellow catfish (*Pelteobagrus fulvidraco*) [30, 31], Nile tilapia (*Oreochromis niloticus L*.) [32], hybrid tilapia (*Oreochromis niloticus* × *Oreochromis mozambique*) [33], different varieties of common carp (*Cyprinus carpio*) [34–36], and grass carp (*Ctenopharyngodon idellus*) [37, 38]. There is great inter specific variation regarding the potential use of BSFLM as future protein substitute, therefore new studies should be performed to identify the most suitable dietary regime for different species.

Carps make an enormous contribution to global aquaculture production, in particular grass carp, common carp, silver carp, bighead carp, rohu and catla which are among the top ten most cultured fish species in the world [1]. Soybean meal is the major protein source for carp feed in Asian aquaculture. A few studies have investigated the effects of replacement of fishmeal by BSFLM in carps [34–36], however, there are very limited studies available on substitution of SBM by BSFLM in carps [37, 38]. To our knowledge, no previous study has added BSFLM to basal diet as substituent of SBM in rohu and catla. Therefore, present study investigated the potential of BSFLM as an alternative of SBM in rohu and catla, which are economically very important species in Asia (the largest fish producing region in the world). The study determined the effects of replacement of crude protein from SBM up to varying levels (0%, 40%, 80%, 100%) with BSFLM on growth performance, whole body chemical composition, profile of amino acids and fatty acids, haematological and biochemical parameters, liver enzymes, oxidative stress biomarkers and disease resistance against one of the most prevalent pathogens (*Staphylococcus aureus*) in these species.

## 2. Materials and methods

### 2.1 Preparation of formulated diets

Four different diets were formulated to be isonitrogenous (23% crude protein). The basal feed formulation has soybean meal (USA) as major protein source. Dried BSFL were procured from KADAP Go Organic, Denmark, larvae were fed on vegetable and fruits waste. In prepared diets, crude protein from SBM was replaced by 0% (T0), 40% (T40), 80% (T80) and 100% (T100) by BSFLM (Table 1). All raw materials were pulverized to 80 mesh. Dicalcium phosphate, methionine, lysine, L-threonine, Axtra^®^ XAP, and microtech 40% were added to pulverized raw materials as per formulation for carps. All these ingredients and the required volume of water were thoroughly mixed in mixer and extruded (CY-3000 extrusion unit, Germany) to make pellets of 3 mm in diameter. Prepared diets were air dried for 24 hours (hrs) and stored at 4°C in sealed bags. Composition of ingredients in experimental diets is given in Table 1.

**Table 1:** Feed formulation of four experimental diets (23% crude protein).

Ingredients (%)	T0 Diet	T40 Diet	T80 Diet	T100 Diet
Maize	39.00	39.00	39.00	39.00
Rice Polish	8.00	8.00	8.00	8.00
Wheat Bran	15.00	15.00	15.00	15.00
APC	5.00	5.00	5.00	5.00
Canola Meal	5.00	5.00	5.00	5.00
Soybean Meal	23.45	14.17	4.07	0.00
BSFL Meal	0.00	10.00	20.00	25.00
Dicalcium Phosphate	1.17	1.10	1.03	0.96
Microtech 10% SD	0.15	0.15	0.15	0.15
Axtra^®^ XAP 10% SD	0.15	0.15	0.15	0.15
DL-Methionine	0.49	0.35	0.29	0.25
Lysine SO_4_ 70%	1.32	1.09	0.95	0.85
L-threonine	0.87	0.59	0.52	0.47
Supplement	0.40	0.40	0.40	0.40
Total	100.00	100.00	100.00	100.00

### 2.2 Growth experiment

Ethical approval was obtained from the Animal Ethics Committee of Lahore College For Women University before performing this trial (Zoo/LCWU/473-479). Fingerlings of juvenile rohu (initial weight = 27.50±0.30 g, n = 130) and catla (initial weight = 16.50±0.53 g, n = 130) were procured from a local hatchery. They were given brine (0.5%) and potassium permanganate (2 mg/L) baths and acclimatized for two weeks prior to the trial in four 700 L fiberglass stock tanks. They were fed with commercial feed (crude protein, 23.43%; moisture, 11.17%; ash, 7.40%; crude fat, 5.67%; crude fiber, 3.97%; starch, 23.00%) during acclimatization period. They were fasted for 24 hours before being distributed to 90 L acrylic aquaria for the trial. Each aquarium has separate water and air supply. Solid waste was removed by water filters and water was exchanged at rate of 10% every day. The total body weight and total body length of fingerlings were measured. Fingerlings of rohu and catla were divided into four groups (T0, T40, T80, T100). The stocking density in each group was 30 fingerlings while each group had three replicates. An additional ten fish were fed with T0 diet to be used for bacterial challenge test as T0+ve group at the end of the growth experiment. Same husbandry protocol was followed for this group as well.

The growth experiment was conducted for 8 weeks to compare the performance of each species fed at 4 replacement levels (n = 3). At the end of the experiment, ten fish per replicate were removed for further analysis for each experimental diet. The trial started on April 20, 2022 and continued for eight weeks. Fish (rohu and catla) in all dietary treatments were hand fed twice a day (8:00 and 16:00 hrs) at a ration rate of 2% of total biomass in each aquarium which is standard practice in carp culture. Fish were reared at ambient temperature and photoperiod. The average water temperature, dissolved oxygen, and pH was 28.00±0.40°C, 2.30±0.06 mg/dL, 7.50±0.30, respectively. Water quality parameters were checked on daily basis by using meters (Hannah, USA) and water quality testing kits (API, USA). Weight check was performed on a weekly basis to adjust the feed ration in all groups.

### 2.3 Sampling

After eight weeks of growth experiment, the fish were not fed for 24 hrs before final sampling. A total of ten fish were randomly selected from each dietary group for bacterial challenge test. The remaining twenty fish in each group were anesthetized by clove oil (6 ml/L) (Sigma Aldrich) and sacrificed after blood collection. Blood was collected from fish gills and hearts. Blood samples were collected into two sets of tubes from each replicate of all four groups of both species. Tubes in one set were coated with sodium heparin, which was used for hematology and assays of catalase (CAT), superoxide dismutase (SOD), and malondialdehyde (MDA). Glass tubes in the second set were without anticoagulant coating. Both sets were stored at 4°C. The second set was centrifuged at 15000 rpm for 20 min at room temperature to collect serum for biochemical analysis. Above mentioned protocol was followed both for rohu and catla. The total body weight (±0.1 g) and total body length (±0.1 cm) of each fish were measured. These data were used to calculate condition factor (%), specific growth rate (%), weight gain (g), weight gain/fish/day (g) and feed conversion ratio (FCR). Fish head and tail were removed in all samples and stored at -4°C for proximate analysis and determining the profile of amino acids and fatty acids.

### 2.4 Haematology, biochemistry, and enzymes analyses

Haemoglobin (g/dl), WBCs (10^3^/μL), RBCs (10^6^/μL), MCV (FL), HCT (%), platelets (10^3^/μL), MCH (%), MCHC (%), neutrophils (%), lymphocytes (%), monocytes (%), eosinophils (%), were measured by using clinical haematology analyser (Sysmex, China). Blood glucose (mg/dl) was measured by laboratory blood glucose analyser. ELISA was performed to measure cholesterol (mg/dl) (Biocompare, USA), and triglycerides (mg/dl) (Abcam, UK) as per manufacturer’s protocol. Alanine aminotransferase (ALT) (U/L), and aspartate aminotransferase (AST) (U/L) were analysed using kits (Thermo Fisher Scientific, USA) on a clinical chemistry analyser (Thermo Fisher Scientific). The assays of CAT (Invitrogen, USA), SOD (Nanjing Pars Biochem, China), and MDA (Nanjing Pars Biochem, China), were performed as per manufacturer’s instructions. Lysozyme activity was measured with assay kit (Nanjing Jiancheng Bioengineering Inc., China) as per given protocol.

Chemical composition of was analysed according to protocol of the Association of Official Analytical Chemists (AOAC, 2005). Whole body samples were dried in oven at 70 ^o^C until constant dry weight was noted. These dry samples were ground for chemical analysis. Crude protein was determined by Kjeldahl apparatus (PCSIR, Pakistan). Total lipids were determined by following Folch method [39] in the Soxhlet apparatus (PCSIR, Pakistan). Samples were incinerated in a muffle furnace (PCSIR, Pakistan) at 560 ^o^C for 4 hrs to determine the ash content from weight loss.

To determine amino acids, 200 mg of each sample was oxidized with 5ml of performic acids in media bottle which were kept at 4°C for 16 hours. For acid hydrolysis of these oxidized samples, 840 mg sodium bisulfite was added to the sample and stirred for ten minutes [40]. A total of 25 ml of 6M HCL was added to sample, stirred for ten minutes, and kept in the oven at 110°C for 24 hours. After acid hydrolysis, pH was adjusted to 2.2 using 7.5M NaOH. Samples were filtered and stored in glass vials for amino acids analysis. The profile of essential amino acids (EAA) and non-essential amino acids (NEAA) was determined in hydrolyzed samples by using the amino acids analyser (Biochrome 30+, UK) (mg/kg). Total lipids collected from whole body samples were esterified by using BF_3_ plus methanol and hexane as solvents. The profile of fatty acids was determined in these esterified samples by a capillary gas chromatograph model Agilent 6890 (USA Agilent Technology) equipped with a split-splitless injector, flame ionization detection (FID) system at PCSIR laboratories, Pakistan.

### 2.5 Bacterial challenge test

Before final sampling (end of growth experiment), a total of ten fish were randomly selected from T0, T40, T80, T100 for bacterial challenge. Fish from T0 was studied as negative (T0-ve) control. As mentioned in section 2.2, an additional ten fish were fed with T0 diet and kept under the same husbandry protocol. These fish were used as positive control (T0+ve) and infected with pathogen. These fish were acclimatized for the next three days after final sampling and fed with relevant experimental diets. Each group (T0+ve, T0-ve, T40, T80, T100) was studied in two replicates while each replicate has five fish. The same protocol was used for both species. Isolate of *Staphylococcus aureus* was obtained from microbiology laboratory of University of Veterinary and Animal Sciences, Pakistan. Bacteria were cultured in nutrient broth 48 hrs prior to trial. A total of 200 μL bacterial culture was added to 250 ml of nutrient broth and incubated overnight at 37°C. The required dose of viable bacterial count of 1×10^8^ CFU/ml [41] was prepared in 5 ml of PBS at optical density of 0.5 and 600 nm wavelength.

To administer bath, 200 ml of prepared bacterial dose was added to 20 L of water in five acrylic aquaria [42]. The final concentration of bacteria in bath was 0.80 CFU/ml. Fish from T0+ve, T40, T80 and T100 were bathed for 2 hrs, provided with continuous aeration. Fish in T0-ve group were bathed in water containing only sterile broth for 2 hrs. After bath, fish were transferred in clean water (90 L acrylic aquaria) to be monitored for next 15 days. Each group in both species was fed with its relevant experimental diet twice a day until satiation. Fish in all groups were monitored for clinical signs of infection like redness of mouth and fins, lesions on skin, lethargy, and mortality. Fish in the terminal stage were killed with an overdose of benzocaine chloride and registered as dead. After 15 days of challenge, fish were collected from each group to collect blood samples as mentioned in section 2.3. These blood samples were used to measure the levels of SOD, CAT and MDA as described in section 2.4.

### 2.6 Statistical analyses

All data were tested for homogeneity of variances by using the Levene’s test. Polynomial orthogonal contrasts test in one-way ANOVA was performed to determine whether the data followed a linear and/or quadratic response to replacement levels of BSFLM with a 95% significance level (*P* < 0.05). If significant effect of treatment was observed, then Tukey’s post hoc test was used to assess the significant differences among means. The parameters which showed insignificant variance after Levene’s test have been mentioned with superscript (^**a**^) for all groups. Scatter plot was drawn for growth parameters to determine if there is a linear plot. In case of linearity, linear regression was performed. The parameters which showed parabola e.g. condition factor and FCR, they were analyzed by quadratic regression. Final values of total body weight, total body length, total biomass, and condition factor were used for regression analysis. The difference was considered as significant when *P* ≤ 0.05. All data are presented as means and standard error (SE). Results were analyzed using the IBM SPSS 22.

## 3. Results

### 3.1 Growth parameters

A significant difference (*P*<0.05) was observed in all growth parameters in rohu except the survival rate which was same (100%) in all four dietary groups (Table 2). Total body weight gradually increased with an increase in inclusion percentage of BSFLM in diet (T0<T40<T80<T100). The maximum increase in weight was observed in fish fed with T100 diet (924.36 g). The same pattern (*P*<0.05) was noted in weight gain/fish/day showing that individual fish gained the maximum weight (0.51 g/day) in T100. Specific growth rate also gradually increased (*P*<0.05) with increase in quantity of BSFLM in diet. All four groups showed good FCR, but it significantly (*P*<0.05) improved in T40, T80 and T100 as compared to T0. The best FCR was noted in T100 (0.75). Condition factor was found to be significantly different (*P*<0.05) between all four groups.

**Table 2:** Growth parameters in rohu (*Labeo rohita*) and catla (*Cala catla*) after feeding with four different experimental diets. Regression analysis was performed for final values only to determine the effect of treatment. Different superscripts across the rows represent the significant variance between treatments calculated by Tukey’s post hoc test.

Growth Parameters	T0	T40	T80	T100	Regression	
					R^2^	*p*
***Labeo rohita***						
Total body weight (g)	35.33±0.53^**a**^	42.00±0.49^**b**^	52.60±0.45^**c**^	58.40±0.39^**d**^	0.988**	0.000
Total body length (cm)	13.26±0.18^**a**^	14.05±0.17^**b**^	14.90±0.16^**c**^	15.65±0.12^**d**^	0.999**	0.000
Condition factor (%)	1.54±0.07^**b**^	1.54±0.06^**b**^	1.61±0.05^**c**^	1.53±0.03^**a**^	0.410*	0.093
Weight gain/fish/day (g)	0.14±0.02^**a**^	0.25±0.03^**b**^	0.42±0.03^**c**^	0.51±0.02^**d**^	0.987**	0.000
Specific growth rate (%)	9.68±1.21^**a**^	10.16±1.36^**b**^	11.03±1.42^**c**^	11.40±1.51^**d**^	0.977**	0.000
Survival rate (%)	100±0.00	100±0.00	100±0.00	100±0.00	NS	NS
Feed conversion ratio	1.38±0.21^**c**^	1.43±0.36^**d**^	0.86±0.02^**b**^	0.75±0.10^**a**^	0.836*	0.010
***Catla catla***						
Total body weight (g)	28.50±0.91^**a**^	28.80±0.39^**b**^	30.20±0.56^**c**^	33.50±0.41^**d**^	0.855**	0.000
Total body length (cm)	9.90±0.06^**a**^	10.72±0.03^**b**^	10.85±0.03^**c**^	11.20±0.05^**d**^	0.894**	0.000
Condition factor (%)	2.93±0.06^**d**^	2.34±0.04^**a**^	2.37±0.05^**b**^	2.39±0.04^**c**^	0.917*	0.000
Weight gain/fish/day (g)	0.21±0.1^**a**^	0.21±0.1^**a**^	0.22±0.03^**b**^	0.28±0.02^**c**^	0.712**	0.001
Specific growth rate (%)	10.36±1.26^**c**^	9.88±1.40^**a**^	10.00±1.62^**b**^	10.36±1.00^**c**^	0.882**	0.000
Survival rate (%)	100±0.00	100±0.00	100±0.00	100±0.00	NS	NS
Feed conversion ratio	0.72±0.01^**a**^	0.83±0.03^**d**^	0.78±0.03^**c**^	0.74±0.02^**b**^	0.796*	0.001

* quadratic regression

**linear regression, NS: analysis could not be performed as all values were same.

Catla also showed good response to replacement of crude protein from SBM with BSFLM (Table 2). However, this species did not respond as good as noted in rohu. Similar to rohu, body weight gradually increased with an increase in inclusion percentage of BSFLM in diet (T0<T40<T80<T100) but numerical difference was not large. A significant difference was observed (*P*<0.05) in specific growth rate, and weight gain/fish/day across all groups. Condition factor also showed a significant different (*P*<0.05) between all four dietary groups. Similarly, FCR in all four groups was significantly different (*P*<0.05) but this parameter did not show any large numerical difference opposite to that observed in rohu. The survival rate was noted to be 100% in all four groups.

### 3.2 Chemical composition of formulated diets and whole body samples

The chemical compositions of all experimental diets were similar (*P*>0.05) in moisture, crude protein, and ash content (Table 3). The concentration of crude fat significantly (*P*<0.05) and linearly increased with increase in quantity of BSFLM in experimental diets. The highest value of crude fat was noted in the T100 diet. The values of moisture, crude protein, crude fat, and ash were observed to be significantly different (*P*<0.05) in whole body chemical composition of both rohu and catla (Table 3). However, numerically, there is no large difference between the values of the same parameter in all four groups of same species. Crude protein content was measured to be numerically higher in rohu as compared with catla while the maximum value was noted in T100 (17.28±0.55%) in rohu.

**Table 3:** Chemical composition (%) (Mean ± SE) of prepared experimental diets and whole body fish samples of rohu (*Labeo rohita*) and catla (*Catla catla*) in all four experimental groups at end of growth experiment. Different superscripts across the rows represent the significant variance between treatments calculated by Tukey’s post hoc test.

Amino acids	T0	T40	T80	T100	BSFLM
**Essential Amino Acids (EAA)**					
Methionine	25.68±0.61^c^	23.89±0.59^a^	24.94±0.59^b^	26.43±0.60^d^	79.12±0.45
Threonine	46.30±0.48^d^	39.60±0.49^a^	42.08±0.48^b^	43.85±0.48^c^	65.92±0.58
Valine	764.34±0.60^a^	846.51±0.62^c^	803.64±0.65^b^	875.72±0.62^d^	1445.59±0.34
Isoleucine	1238.81±0.29^b^	1209.32±0.27^a^	1310.96±0.27^d^	1251.15±0.28^c^	1516.72±0.18
Leucine	8.91±0.11^a^	9.33±0.11^b^	10.23±0.13^d^	9.76±0.11^c^	5.41±0.61
Phenylalanine	225.77±0.59^a^	308.02±0.57^d^	271.04±0.56^c^	235.04±0.57^b^	69.61±0.37
Histidine	162.24±0.28^b^	154.29±0.21^a^	162.25±0.20^b^	165.35±0.29^c^	332.31±0.29
Lysine	41.95±0.30^d^	39.11±0.29^a^	39.86±0.30^b^	40.99±0.31^c^	57.73±0.52
Tryptophan	6.41±0.19^c^	6.41±0.17^c^	6.20±0.17^b^	6.08±0.18^a^	1.28±0.38
TEAA	2520.41	2636.48	2671.20	2654.37	3573.69
**Non Essential Amino Acids (NEAA)**					
Asparagine	87.55±0.60^c^	75.57±0.59^a^	75.70±0.57^b^	87.85±0.58^d^	106.72±0.67
Glutamine	25.39±0.44^b^	24.42±0.45^a^	26.01±0.47^c^	26.42±0.48^d^	20.53±0.61
Alanine	857.98±0.50^b^	768.00±0.51^a^	883.89±0.50^d^	867.91±0.50^c^	1175.42±0.83
Tyrosine	1.89±0.28^b^	1.76±0.25^a^	2.09±0.27^d^	1.99±0.27^c^	2.56±0.18
TNEAA	972.81	869.75	987.69	983.87	1305.23
TSAA	25.68	23.89	24.94	26.43	79.12
TArAA	396.31	470.48	441.58	408.46	405.76

TEAA: Total Essential Amino Acids, TNEAA: Total Non-Essential Amino Acids, TSAA: Total Sulfur Amino Acids, TArAA: Total Aromatic Amino Acids

### 3.3 Amino acids profile in formulated diets, BSFLM, and whole body samples

The profile of amino acids in prepared experimental diets (T0, T40, T80, T100) showed the significant difference (*P*<0.05) (Table 4). The values of EAA and NEAA in BSFLM were noted to be quantitatively higher than those in experimental diets except tryptophan. Although significantly different (*P*<0.05) but quantitatively, the profile of both EAA and NEAA did not show high variations between four experimental diets.

**Table 4:** Determination of essential and non-essential amino acids (Mean ± SE) (mg/kg) in experimental diets. Different superscripts across the rows represent the significant variance between treatments calculated by Tukey’s post hoc test.

Experimental Diets				
Contents	T0	T40	T80	T100
Moisture	11.08±0.25^a^	11.10±0.52^a^	11.13±0.63^a^	11.11±0.14^a^
Crude Protein	23.09±1.03^a^	23.11±1.13^a^	23.10±1.04^a^	23.00±1.15^a^
Crude Fat	6.28±0.41^a^	6.25±0.52^a^	6.30±0.33^a^	6.26±0.48^a^
Crude Ash	6.09±0.36^a^	6.12±0.53^a^	6.18±0.51^a^	6.27±0.50^a^
***Labeo rohita***				
Moisture	76.51±0.65^a^	76.62±0.66^b^	78.51±0.65^d^	77.89±0.65^c^
Crude Protein	16.84±0.54^c^	15.81±0.54^a^	16.16±0.55^b^	17.28±0.55^d^
Crude Fat	2.30±0.55^c^	3.63±0.57^b^	3.65±0.57^a^	3.75±0.57^b^
Crude Ash	2.14±0.47^c^	2.50±0.49^d^	2.82±0.47^b^	2.76±0.50^a^
***Catla catla***				
Moisture	78.03±0.60^b^	79.51±0.60^d^	77.65±0.60^a^	79.25±0.60^c^
Crude Protein	15.44±0.65^d^	14.82±0.61^a^	15.40±0.65^c^	15.29±0.65^b^
Crude Fat	2.28±0.53^c^	3.46±0.54^a^	3.63±0.54^b^	3.77±0.53^c^
Crude Ash	2.20±0.54^b^	2.32±0.54^b^	2.88±0.54^c^	2.62±0.54^a^

In rohu and catla, values of all EAA and NEAA were significantly different (*P*<0.05) between all dietary groups (Table 5). However, these values were observed to be numerically similar between these groups. Comparatively, the profile of amino acids in rohu were found to be higher than those in catla.

**Table 5:** Determination of essential and non-essential amino acids (mg/kg) (Mean ± SE) in whole body samples of rohu (*Labeo rohita*) and catla (*Catla catla*) at end of growth experiment. Different superscripts across the rows represent the significant variance between treatments calculated by Tukey’s post hoc test.

Essential Amino Acids (EAA)								
	*Labeo rohita*				*Catla catla*			
Amino Acids	T0	T40	T80	T100	T0	T40	T80	T100
Methionine	112.67±0.21^a^	112.70±0.20^a^	114.00±0.18^b^	114.13±0.21^b^	85.66±0.35^a^	85.76±0.30^a^	87.35±0.35^b^	87.38±0.33^b^
Threonine	97.53±0.31^c^	85.44±0.28^a^	96.75±0.28^b^	96.87±0.27^b^	132.78±0.23^d^	108.66±0.21^a^	110.19±0.22^b^	126.66±0.22^c^
Valine	2802.55±0.27^d^	2355.06±0.27^a^	2568.25±0.29^b^	2641.90±0.28^c^	2505.13±0.30^d^	2145.78±0.27^a^	2180.93±0.27^b^	2381.38±0.29^c^
Isoleucine	3769.41±0.34^d^	3275.73±0.30^a^	3676.67±0.33^c^	3661.41±0.33^b^	3756.60±0.63^d^	3046.47±0.61^a^	3292.78±0.63^b^	3742.04±0.62^c^
Leucine	2.37±0.57^a^	2.41±0.60^b^	2.48±0.59^c^	2.49±0.60^c^	144.46±0.45^c^	125.00±0.45^a^	133.29±0.47^b^	149.56±0.45^d^
Histidine	686.39±0.38^d^	510.63±0.39^a^	591.94±0.38^c^	577.91±0.36^b^	692.08±0.59^d^	506.59±0.62^a^	572.56±0.60^b^	677.98±0.60^c^
Lysine	165.84±0.27^d^	106.78±0.30^a^	127.93±0.32^c^	108.02±0.32^b^	173.86±0.61^c^	146.92±0.57^a^	155.93±0.57^b^	175.92±0.59^d^
Ornithine	13.29±0.01^a^	13.31±0.01^a^	13.45±0.02^b^	13.58±0.0 1^c^	13.11±0.07^c^	12.85±0.06^a^	13.08±0.07^b^	13.43±0.06^d^
TEAA	7650.05±0.30	6462.06±0.29	7191.47±0.30	7216.31±0.30	7503.68±0.40	6178.03±0.39	6546.11±0.40	7354.35±0.40
**Non Essential Amino Acids (NEAA)**								
Cysteine	2.44±0.02^a^	2.98±0.02^b^	3.73±0.02^c^	3.74±0.02^c^	0.41±0.03^c^	0.33±0.02^a^	0.40±0.03^bc^	0.38±0.03^b^
Aspartic Acid	6.20±0.77^a^	9.50±0.75^c^	10.08±0.76^d^	9.04±0.75^b^	11.90±0.63^c^	11.33±0.67^a^	11.58±0.66^b^	12.12±0.67^d^
Serine	300.95±0.58^a^	300.95±0.54^a^	302.00±0.58^b^	304.00±0.57^c^	358.31±0.60^a^	358.33±0.60^a^	368.82±0.61^b^	389.84±0.60^c^
Glutamic Acid	45.48±0.94^a^	46.66±0.97^b^	47.25±0.92^c^	47.69±0.97^d^	25.84±0.71^b^	25.69±0.71^a^	25.98±0.73^c^	26.28±0.71^d^
Glycine	57.21±0.31^b^	53.76±0.31^a^	57.82±0.33^c^	58.20±0.31^d^	53.57±0.62^d^	48.71±0.60^a^	48.86±0.60^b^	50.26±0.61^c^
Alanine	2906.75±0.23^d^	2365.96±0.21^a^	2503.70±0.22^b^	2503.79±0.22^c^	2729.57±0.31^d^	2198.93±0.35^a^	2401.40±0.36^b^	2726.97±0.30^c^
Tyrosine	5.90±0.01^c^	6.63±0.03^d^	3.51±0.01^b^	3.35±0.01^a^	4.38±0.03^a^	4.49±0.03^b^	4.95±0.03^c^	5.03±0.03^d^
TNEAA	3324.93±0.41	2786.44±0.40	2928.09±0.41	2929.81±0.41	3183.98±0.42	2647.81±0.43	2861.99±0.43	3210.88±0.42
TSAA	115.11±0.11	115.68±0.11	117.73±0.10	117.87±0.11	86.07±0.19	86.09±0.16	87.75±0.19	87.76±0.18
TArAA	692.29±0.20	517.26±0.21	595.45±0.20	581.26±0.19	696.46±0.31	511.08±0.33	577.51±0.32	683.01±0.32

TSAA: Total Sulfur Amino Acids, TArAA: Total Aromatic Amino Acids

### 3.4 Fatty acids profile in formulated diets, BSFLM, and whole body samples

The profile of fatty acids was observed to be significantly different (*P*<0.05) between all four diets (Table 6). However numerically, these values were similar between all four diets except lauric acid, myristic acid, eicosapentanoic acid (EPA), and decosahexanoic acid (DHA). The values of these four fatty acids increased with an increase in proportion of BSFLM in diet while their highest concentration was noted in BSFLM. Similarly, n3:n6 fatty acids ratio also gradually increased (T0<T40<T80<T100) in diets.

**Table 6:** Determination of fatty acids (Mean ± SE) in total lipids extracted from experimental diets. The values are expressed as percentage of total fatty acids. Different superscripts across the rows represent the significant variance between treatments calculated by Tukey’s post hoc test.

Saturated Fatty Acids (SFA)						
C: D	Common Name	T0	T40	T80	T100	BSFLM
C12:0	Lauric Acid	1.34±0.16^a^	3.69±0.18^b^	6.42±0.17^c^	8.10±0.18^d^	28.60±0.33
C14:0	Myristic Acid	0.44±0.07^a^	0.58±0.09^b^	0.62±0.07^c^	0.65±0.08^d^	6.10±0.31
C16:0	Palmitic Acid	26.20±1.53^d^	24.71±1.61^c^	23.90±1.58^b^	23.13±1.64^a^	12.60±0.41
C18:0	Stearic Acid	3.80±1.19^d^	3.21±1.25^c^	2.98±1.29^a^	3.01±1.09^b^	2.20±0.20
C20:0	Arachidic Acid	1.10±0.17^a^	1.32±0.20^c^	1.43±0.15^d^	1.28±0.16^b^	0.00±0.00
C22:0	Docosanoic Acid	0.45±0.09^a^	0.51±0.09^bc^	0.53±0.07^c^	0.50±0.08^b^	0.00±0.00
C24:0	Lignoceric Acid	0.20±0.15^a^	0.27±0.19^b^	0.22±0.15^a^	0.25±0.16^b^	0.00±0.00
Total SFA		33.53	34.29	36.10	36.92	49.50
**Monounsaturated Fatty Acids (MUFA)**						
C16:1 n-6	Sapienic Acid	0.11±0.53^a^	0.13±0.60^a^	0.32±0.35^c^	0.27±0.43^b^	4.80±0.41
C18:1 n-9	Oleic Acid	36.50±1.86^d^	31.93±1.97^a^	32.05±1.98^b^	34.84±1.98^c^	25.12±0.42
Total MUFA		36.61	32.06	32.37	35.11	29.92
**Polyunsaturated Fatty Acids (PUFA)**						
C18:2 n-6	Linoleic Acid	19.87±0.99^d^	18.33±0.98^b^	17.87±0.77^a^	19.22±0.83^c^	12.52±0.18
C18:3 n-3	α-linolenic Acid	4.46±0.07^d^	4.12±0.07^c^	3.99±0.08^a^	4.02±0.08^b^	3.40±0.27
C20:5 n-3	Eicosapentanoic Acid	0.00±0.15^a^	0.92±0.07^b^	0.96±0.06^c^	0.94±0.01^bc^	1.70±0.01
C22:6 n-3	Decosahexanoic Acid	0.00±0.39^a^	0.58±0.00^b^	0.62±0.02^c^	0.63±0.03^c^	0.70±0.09
Total PUFA		24.33	23.95	23.44	24.81	18.32
Total UFAs		60.94	56.01	55.81	59.92	48.24
n3:n6		0.22	0.31	0.31	0.29	0.46

The profile of fatty acids in both rohu and catla were significantly (*P*<0.05) different between all four groups (Table 7). However, these values were numerically similar except in α-linolenic acid and DHA which increased with increase in quantity of BSFLM in diet (T0<T40<T80<T100) in both species. The value of lauric acid gradually increased and found to be maximum in T100 both in rohu and catla. Similarly, n3:n6 fatty acids ratio also gradually increased (T0<T40<T80<T100) in both species.

**Table 7:** Determination of fatty acids (Mean ± SE) in total lipids extracted from whole body samples of rohu (*Labeo rohita*) and catla (*Catla catla*) at end of growth experiment. Values are expressed as percentage of total fatty acids. Different superscripts across the rows represent the significant variance between treatments calculated by Tukey’s post hoc test.

Saturated Fatty Acids (SFA)									
		*Labeo rohita*				*Catla catla*			
C: D	Common Name	T0	T40	T80	T100	T0	T40	T80	T100
C12:0	Lauric Acid	1.41±0.17^a^	1.47±0.17^b^	1.50±0.18^c^	1.80±0.19^d^	0.40±0.31^a^	0.61±0.33^b^	0.64±0.31^c^	0.70±0.30^d^
C14:0	Myristic Acid	2.50±0.08^b^	2.20±0.08^a^	2.50±0.08^b^	2.70±0.09^c^	3.30±0.34^a^	4.50±0.31^d^	3.50±0.29^b^	3.80±0.34^c^
C16:0	Palmitic Acid	20.30±1.54^a^	22.80±1.60^c^	22.00±1.59^b^	23.10±1.65^d^	20.70±1.14^a^	21.20±1.29^b^	22.30±1.31^d^	22.00±1.13^c^
C18:0	Stearic Acid	8.50±1.20^b^	8.70±1.24^c^	9.50±1.30^d^	7.80±1.20^a^	10.30±0.32^a^	12.00±0.32^d^	11.50±0.38^c^	10.80±0.34^b^
C20:0	Arachidic Acid	1.37±0.18^b^	1.80±0.19^d^	1.20±0.16^a^	1.40±0.17^c^	0.80±0.23^a^	1.10±0.31^b^	1.20±0.42^c^	1.10±0.32^b^
C22:0	Docosanoic Acid	0.80±0.09^a^	2.60±0.08^c^	2.50±0.08^b^	2.70±0.09^d^	0.70±0.28^a^	1.10±0.33^c^	0.80±0.33^b^	1.10±0.41^c^
C24:0	Lignoceric Acid	0.40±0.18^c^	0.60±0.18^d^	0.10±0.16^a^	0.30±0.17^b^	0.30±0.22^b^	0.20±0.32^a^	0.70±0.40^c^	0.30±0.34^b^
Total SFA		35.28	40.17	39.30	39.80	36.50	40.71	40.64	39.80
**Monounsaturated Fatty Acids (MUFA)**									
C16:1 n-6	Sapienic Acid	3.50±0.54^d^	3.20±0.61^c^	2.70±0.36^a^	3.00±0.44^b^	2.03±0.21^a^	2.60±0.42^d^	2.30±0.36^b^	2.50±0.40^c^
C18:1 n-9	Oleic Acid	39.70±1.87^a^	40.90±1.98^b^	42.60±1.99^c^	43.30±1.99^d^	40.40±1.32^a^	44.20±1.43^c^	43.50±1.39^bc^	44.50±1.47^d^
Total MUFA		43.20	44.10	45.30	46.30	42.43	46.80	45.80	47.00
**Polyunsaturated Fatty Acids (PUFA)**									
C18:2 n-6	Linoleic Acid	17.70±1.00^d^	10.20±0.99^c^	8.80±0.78^b^	8.60±0.64^a^	18.00±1.10^d^	9.13±1.19^c^	8.50±1.07^b^	8.40±1.04^a^
C18:3 n-3	α-Linolenic Acid	2.80±0.08^b^	2.70±0.08^a^	3.50±0.09^d^	3.30±0.09^c^	1.30±0.34^a^	1.50±0.28^b^	2.60±0.27^d^	2.20±0.31^c^
C20:5 n-3	Eicosapentanoic Acid	1.10±0.16^a^	1.80±0.48^d^	1.20±0.17^b^	1.40±0.18^c^	0.80±0.19^a^	1.10±0.30^b^	1.50±0.34^d^	1.40±0.27^c^
C22:6 n-3	Decosahexanoic Acid	0.80±0.19^a^	2.60±0.35^c^	2.50±0.34^b^	2.62±0.35^c^	0.40±0.10^a^	0.80±0.19^b^	1.20±0.20^c^	1.30±0.25^d^
Total PUFA		22.40	17.30	16.00	15.92	20.50	12.53	13.80	13.30
Total UFAs		65.60	61.40	61.30	62.22	62.93	59.33	59.60	60.30
n3:n6		0.27	0.70	0.82	0.85	0.14	0.37	0.62	0.58

### 3.5 Hematological and biochemical parameters

The values of haemoglobin, WBC, RBCs, MCV, HCT, platelets, MCH, MCHC, neutrophils, lymphocytes, monocytes, eosinophils were observed to be significantly different (*P*<0.05) among all four dietary groups in both rohu and catla (Table 8). However, these values were numerically similar in all these dietary groups. Similar results were noted in the case of blood glucose in both species as well. The levels of cholesterol gradually increased in T40, T80 and T100 as compared to that in T0 in both species. The highest value of triglyceride was noted in T100 in both rohu and catla. The concentrations of ALT and AST were significantly different (*P*<0.05) but numerically similar among all four dietary groups in both species.

**Table 8:** Determination of haematological and biochemical parameters (Mean ± SE) in rohu (*Labeo rohita*) and catla (*Catla catla*) at end of growth experiment. Different superscripts across the rows represent the significant variance between treatments calculated by Tukey’s post hoc test.

Hematological Parameters								
	*Labeo rohita*				*Catla catla*			
Parameters	T0	T40	T80	T100	T0	T40	T80	T100
Hemoglobin (g/dl)	5.80±0.27^b^	3.40±0.24^a^	3.40±0.24^a^	3.40±0.27^a^	5.60±0.41^c^	6.40±0.41^d^	4.25±0.41^a^	5.55±0.41^b^
WBCs (10^3^/μL)	4.75±0.82^d^	3.60±0.79^b^	4.25±0.81^c^	2.50±0.74^a^	3.10±0.82^a^	3.60±0.82^b^	3.60±0.82^b^	3.10±0.81^a^
RBCs (10^6^/μL)	1.88±0.08^d^	1.50±0.06^b^	1.80±0.07^c^	1.45±0.06^a^	2.10±0.82^c^	2.10±0.82^c^	1.75±0.82^b^	1.60±0.82^a^
MCV (FL)	78.50±0.66^a^	88.00±0.69^d^	83.83±0.65^c^	83.33±0.65^b^	98.00±0.87^c^	110.00±0.87^d^	88.30±0.87^b^	85.00±0.87^a^
HCT (%)	12.95±0.82^d^	8.60±0.79^b^	9.55±0.80^c^	8.10±0.78^a^	13.60±0.82^c^	14.60±0.82^d^	9.60±0.82^a^	10.75±0.82^b^
Platelets (10^3^/μL)	240.00±0.20^c^	177.66±0.22^b^	283.00±0.21^d^	127.50±0.22^a^	210.00±0.82^b^	233.00±0.82^c^	310.00±0.82^d^	156.00±0.82^a^
MCH (%)	67.00±0.73^a^	88.00±0.82^d^	76.00±0.80^c^	68.00±0.74^b^	88.00±0.82^d^	78.00±0.82^c^	72.50±0.82^a^	73.00±0.82^b^
MCHC (%)	83.50±0.79^a^	92.00±0.83^d^	87.00±0.81^b^	87.50±0.82^c^	98.00±0.75^c^	98.00±0.75^c^	93.00±0.75^b^	82.66±0.75^a^
Neutrophils (%)	30.50±0.82^d^	30.00±0.81^c^	23.00±0.72^a^	28.00±0.27^b^	30.00±0.8^c^	26.00±0.73^a^	26.00±0.82^a^	28.00±0.78^b^
Lymphocytes (%)	65.00±0.79^a^	66.00±0.80^b^	72.50±0.83^c^	83.33±0.74^d^	66.00±0.8^a^	70.00±0.81^c^	70.00±0.81^c^	68.00±0.81^b^
Monocytes (%)	2.08±0.07^a^	2.50±0.09^b^	2.50±0.09^b^	2.50±0.06^b^	2.10±0.82^a^	2.10±0.82^a^	2.10±0.82^a^	2.10±0.82^a^
Eosinophils (%)	2.08±0.06^b^	2.08±0.06^b^	2.08±0.06^b^	1.45±0.65^a^	2.10±0.82^a^	2.10±0.82^a^	2.10±0.82^a^	2.10±0.82^a^
**Biochemical Parameters**								
Glucose(mg/dl)	80.50±0.81^c^	87.00±0.82^d^	66.50±0.78^a^	78.00±0.80^b^	66.00±0.81^a^	66.00±0.81^a^	67.00±0.81^b^	79.00±0.81^c^
Cholesterol (mg/dl)	163.00±0.79^a^	188.00±0.90^c^	182.67±0.86^b^	188.00±0.90^c^	169.00±0.82^a^	177.00±0.82^b^	181.00±0.82^d^	178.16±0.82^c^
Triglycerides (mg/dl)	168.16±0.70^b^	167.00±0.74^a^	210.00±0.80^d^	185.00±0.76^c^	188.00±0.83^b^	167.00±0.83^a^	204.00±0.83^d^	199.00±0.82^c^
ALT (U/L)	49.50±0.82^c^	44.00±0.79^a^	44.00±0.79^a^	47.16±0.80^b^	56.00±0.81^c^	37.00±0.81^a^	62.00±0.81^d^	47.00±0.81^b^
AST (U/L)	40.00±0.80^b^	32.00±0.77^a^	45.50±0.82^d^	42.00±0.81^c^	44.00±0.81^c^	29.00±0.81^a^	46.00±0.81^d^	35.00±0.81^b^

### 3.6 Oxidative stress biomarkers (before and after bacterial challenge)

The values of CAT, SOD, and MDA observed at end of the growth experiment were found to be significantly different (*P*<0.05) between different dietary groups in both rohu and catla (Table 9). The concentration of CAT and SOD significantly (*P*<0.05) increased with inclusion of BSFLM in diet in both species. However, levels of MDA were significantly different (*P*<0.05) but did not show a large difference between four dietary groups in both species as observed in CAT and SOD. Lysozyme activity significantly (*P*<0.05) increased with an increase in BSFLM in diets of both species (Table 9).

**Table 9:** Determination (Mean ± SE) of catalase (CAT) (U/ml), malondialdehyde (MDA) (nmol/ml) superoxide dismutase (SOD) (ng/ml), and lysozyme (LZM) (U/ml) in rohu (*Labeo rohita*) and catla (*Catla catla*) at end of growth experiment. The values of CAT, SOD and MDA were also measured at end of the bacterial (*Staphylococcus aureus*) challenge test (0.80 CFU/ml). Different superscripts across the columns represent the significant variance between treatments calculated by Tukey’s post hoc test.

Groups	*Labeo rohita*				*Catla catla*			
	CAT	SOD	MDA	LZM	CAT	SOD	MDA	LZM
**End of Feeding Trial**								
T0	2.56±0.17^a^	0.39±0.01^a^	0.60±0.04^a^	130.12±10.12^a^	0.50±0.01^a^	0.33±0.01^a^	0.32±0.01^b^	532.14±20.42^a^
T40	2.84±0.02^b^	0.43±0.02^b^	0.65±0.04^d^	144.70±11.63^b^	0.63±0.01^b^	0.42±0.01^b^	0.35±0.01^d^	546.00±19.36^b^
T80	2.88±0.01^b^	0.47±0.02^c^	0.63±0.02^c^	146.80±7.87b^a^	0.66±0.01^c^	0.49±0.06^c^	0.33±0.01^c^	549.53±21.14^b^
T100	2.94±0.01^c^	0.49±0.02^d^	0.61±0.01^b^	151.50±12.82^c^	0.72±0.01^d^	0.53±0.04^d^	0.31±0.03^a^	561.87±18.21^c^
**End of Bacterial Challenge Test**								
	**CAT**	**SOD**	**MDA**	**Survival (%)**	**CAT**	**SOD**	**MDA**	**Survival (%)**
T0-ve	2.48±0.03^b^	0.38±0.03^a^	0.62±0.01^a^	100.00±0.00^d^	0.51±0.02^b^	0.35±0.01^a^	0.31±0.01^a^	100.00±0.00^c^
T0+ve	0.95±0.01^a^	0.44±0.01^b^	0.71±0.01^c^	55.00±1.00^a^	0.15±0.01^a^	0.36±0.01^a^	0.48±0.01^d^	46.00±1.00^a^
T40	5.64±0.04^c^	0.44±0.02^b^	0.62±0.02^a^	72.00±2.00^b^	0.65±0.07^e^	0.39±0.01^b^	033±0.01^c^	71.00±1.00^b^
T80	5.72±0.03^d^	0.47±0.02^c^	0.64±0.02^b^	75.00±1.00^c^	0.64±0.01^c^	0.42±0.02^c^	0.33±0.02^c^	70.00±2.00^b^
T100	5.81±0.02^e^	0.73±0.03^d^	0.62±0.04^a^	75.00±1.00^c^	0.68±0.06^d^	0.46±0.03^d^	0.32±0.01^b^	70.00±2.00^b^

T0-ve was not infected. T+ve was infected.

***After*** bacterial challenge, a significant (*P*<0.05) difference was noted in values of CAT, SOD and MDA in different treatments groups of both species. The levels of CAT and SOD linearly increased with an increase in proportion of BSFLM in diet. On the hand, the level of MDA in both species significantly increased in T0+ve group but its levels in other groups were closer to the values observed in their relevant treatments at end of the growth experiment. The value of CAT, SOD and MDA in T0-ve were similar to those observed at end of the growth experiment. Infected fish in T0+ve groups of both species showed the lowest (*P*<0.05) levels of CAT and SOD.

In both species, signs of infection started to appear by day-3 of exposure beginning with lethargy. Lesions, skin discoloration and inflammation of the mouth appeared between day-5 –day-7 in both species. Clinical signs of infection appeared in 100% fish in all exposed dietary groups of both species by day-12. In rohu, signs of infection improved by day-14 up to 65% and 100% in T80 and T100, respectively except lethargy. In catla, these clinical signs remained present till end of the trial in all dietary groups exposed to infection. The survival rate in both rohu and catla was 55% and 46% in T0+ve, respectively. Mortality significantly improved with an increase in BSFLM in experimental diets. No mortality was noted in T0-ve groups of both species.

## 4. Discussion

The current study observed notable growth and an improved Feed Conversion Ratio (FCR) in rohu when substituting crude protein from soybean meal (SBM) with black soldier fly larvae meal (BSFLM), with the most favorable outcomes seen at a complete 100% replacement with BSFLM. Conversely, catla displayed similar growth performance and FCR in both groups, whether they were fed with BSFLM or SBM, indicating no adverse effects from incorporating BSFLM. For both species, it is evident that BSFLM provides all the necessary nutrients required for the growth of juvenile rohu and catla. Similar results were observed in different varieties of common carp [34–36], Nile tilapia [32, 43]), hybrid tilapia [33], grass carp [37, 38] and other carnivorous species (meagre: [19], rainbow trout: [17, 18], Atlantic salmon: [20–22], turbot: [11], Siberian sturgeon: [24], Japanese seabass: [23], European sea bass: [27, 28], African catfish: [25]) with partial substitution of BSFLM. However, only two studies [37, 38] replaced SBM with BSFLM which is the main objective of present study as well. Significant improvement in growth parameters and FCR as observed in rohu has been noted only in common carp after addition of BSFL oil to a costly multi meal feed (fishmeal, SBM, rapeseed meal, cottonseed meal, full fat soybean) [44]. This could be attributed to the restricted replacement of actual crude protein (ranging from 0% to 7%) with BSFLM. A higher proportion of BSFLM, and subsequently an increased chitin content, could potentially diminish diet palatability, digestibility of protein, intake of feed, and growth performance in fish [11]. Comparatively lower growth response of catla to BSFLM than that noted in rohu at same inclusion rate may be due to different tolerance levels of insect components among different species [38]. However, replacement of crude protein from SBM with BSFLM up to 100% in diet of rohu and catla is appropriate for good growth and immune response in both species.

The profile of all EAA and NEAA in prepared diets and whole body fish also support these results. In this study, dietary replacement of crude protein from SBM up to 100% did not affect the proximate (chemical) composition and profile of amino acid of whole fish in both species. These findings are consistent with previous studies in common carp [34–36], grass carp [38], Atlantic salmon [45], and barramundi (*Lates calcarifer*) [46]. BSFLM used in present study, contained large quantity of true protein, an excellent source of lipids, and a balanced amino acid profile, similar to that in fishmeal [47, 48]. The amino acid profile of BSFLM surpasses that of SBM and other prevalent plant protein sources [49]. It is larger values of lysine, methionine, threonine, valine and isoleucine as also noted in present study (Table 4) [15].

Profile of most of fatty acids in fish were similar between all four dietary groups both in rohu and catla. However, progressive increase in α-linolenic acid, EPA and DHA was observed with proportional % inclusion of BSFLM in diet of both species. Table 6 shows that the larger part of these fatty acids in fish may be ascribed to BSFLM in the diet and their endogenous biosynthesis. Similar results were reported in grass carp when SBM was replaced with BSFLM [37] in Siberian sturgeon [24] and Atlantic salmon [45] as well. A few other studies have reported the opposite response after replacement of fishmeal with BSFLM in different species like common carp [36], rainbow trout [50], and red seabream (*Pagrus major*) [51]. These differences in findings may be due to variation in species, size, and methods to feed and process insects [52]. The profile of lauric acid also linearly increased with dietary addition of BSFLM in both carps. Similar trends have been observed in several species like common carp [36], Siberian sturgeon [24], Atlantic salmon [45], grass carp [37], and red seabream [51]. Increased values of EPA, DHA and n3:n6 fatty acids ratio after replacement of crude protein from SBM with BSFLM are index of high nutritional value of fish, suggesting that BSFLM enriched the nutritional quality of rohu and catla. Likewise, the elevated levels of lauric acid following the substitution with BSFLM could improve the antibacterial and antiviral defense in these species, particularly effective against gram-positive bacteria [13, 53] as observed in bacterial exposure trial in present study.

Haematological parameters are important criteria to assess the impact of changes in feed ingredients on fish health. The lack of significant differences in these parameters across all four dietary groups in both species indicates their capacity to effectively incorporate BSFLM without compromising their physiological functions such as transportation of O_2_, the capacity to defend against foreign bodies, and overall health status. Similar results have been noted in other species as well (Atlantic salmon; [45]; African catfish; [25]; grass carp; [38]; mirror carp; [44]. The levels of cholesterol and triglycerides slightly increased in all dietary groups in both species as observed in rainbow trout [35], grass carp [38], meagre [19], and Japanese seabass [23]. Comparatively, the lower levels of ALT and AST enzymes in BSFLM fed fish also signify that the addition of BSFLM will not impair the hepatic tissue health and integrity in rohu and catla as observed in other species also [25, 38, 44]. Lysozyme activity in both rohu and catla increased in response to addition in BSFLM as also observed in yellow catfish [31] and European seabass [54]. Improvement in lysozyme activity will stimulate the immune response of fish and may enhance the resistance against pathogens [55]. This finding suggests that BSFLM increased the activity of enzymes related to immunity and enhanced the immune response of rohu and catla. Unfortunately, lysozyme activity could not be measured after bacterial challenge test due to insufficient blood samples.

Alteration in ingredients of fish diet can influence the oxidative stress response in animals. Elevation in reactive oxygen species (ROS) causes hydroxylation of DNA, denaturation of protein, peroxidation of lipids, and cell damage [56]. In the present study, levels of CAT and SOD linearly increased with an increase in BSFLM in diet at the end of growth experiment in both species. Similar results have been reported in Jian carp [35, 36], yellow catfish [31], African catfish [25], mirror carp [44], largemouth bass [26], and European seabass [54]. However, MDA content in both species showed a tendency of similarity with the group fed with zero BSFLM (T0). This finding is in contrast with most of aforementioned species which reported a decrease in MDA after inclusion of BSFLM. Synthesis of MDA is an output of lipid peroxidation especially n-3 PUFA which causes disruption of the membrane lipid bilayer and alters membrane structure and permeability [57]. However, lipid peroxidation is not the sole reason behind elevation in ROS [58]. From these findings, it can be inferred that n-3 PUFA added by BSFLM might have been deposited in tissues/cells instead of being peroxidized during growth experiment of both species.

The present study further investigated the role of BSFLM against disease resistance by experimentally challenging the BSFLM fed fish with *Staphylococcus aureus*, one of the most common pathogens of carps. Survivability of fish fed with BSFLM was higher than T0+ve group (zero BSFLM) in both species. These findings were consistent with studies on European seabass [54], and barramundi [59] but these species were exposed to different pathogens. Levels of CAT and SOD increased in the dietary groups with a higher proportion of BSFLM at end of the challenge test. Opposite to the growth experiment, levels of MDA remained almost similar to those observed at the end of growth experiment and T0-ve control. Similar results were observed both in rohu and catla after pathogenic exposure. These findings may be attributed to identify antimicrobial activity of several peptides and lauric acid present in BSFL against a several gram-positive bacteria including *Staphylococcus aureus* [12, 13, 53, 60]. Moreover, beneficial scavenging activity of chitin present in exoskeleton of insects against free radicals such as ROS has been determined as well [61]. Nonetheless, future research is required to identify the mechanisms of action of these antioxidant materials present in BSFL.

## 5. Conclusion

The findings of present study illustrate that replacement of crude protein from SBM with BSFLM in diet of rohu and catla up to 100% improved the growth, weight gain, and feed conversion ratio. This substitution did not impair the chemical composition, particularly the amino acids and fatty acids profiles which are indices of nutritional quality in fish. Fish health status, antioxidant response and resistance to *Staphylococcus aureus* in both species also indicate that BSFLM is a suitable alternative to SBM in carp feed. However, suitability of BSFLM depends upon the feeding material ingested by larvae during its culture since they are highly responsive to their diet. Moreover, preparation of diet with 100% substitution of BSFLM proved to be challenging at the stage of mixing, sieving, and extrusion due to high fat content. Defatting of BSFLM should be considered for future studies in case of its inclusion higher than 80% of crude protein. Large scale local production of BSFLM will be more economical than importation/production of soybean in Asia, particularly when required crude protein content in carps feed (specifically rohu and catla) is ≤ 25% at all stages of their grow out period at commercial scale.

## Supporting information

S1 Data (XLSX)
